# In vitro treatment of 3 T3-L1 adipocytes with recombinant Calcium/calmodulin-dependent Protein Kinase IV (CaMKIV) limits ER stress and improves insulin sensitivity through inhibition of autophagy via the mTOR/CREB signaling pathway

**DOI:** 10.1186/s12902-020-00589-2

**Published:** 2020-07-13

**Authors:** Jiali Liu, Ruihua Yang, Hao Meng, Ting Zhou, Qian He

**Affiliations:** 1 Department of Clinical Laboratory, Xi’an Jiaotong University Second Affiliated Hospital, 157 West 5 Road, Xi’an, 710004 Shaanxi China; 2 Leonard Davis School of Gerontology, University of Southern California, Los Angeles, CA 90089 USA

**Keywords:** CaMKIV, Adipose, ER stress, Autophagy, Insulin resistance

## Abstract

**Background:**

Recently, CaMKIV has been identified as a potential regulator of skeletal muscle glucose metabolism, it can also affect insulin gene expression in pancreas. However, its effects on adipose insulin resistance have yet to be explored. Autophagy has been shown as a potential therapeutic target for ER (endoplasmic reticulum) stress and insulin resistance. The purpose of this study is to investigate the effects of CaMKIV on ER stress, autophagic function and insulin signaling in tunicamycin-treated adipocytes.

**Methods:**

In this study, mature 3 T3-L1 adipocytes were treated with tunicamycin to induce ER stress. Tunicamycin-treated 3 T3-L1 adipocytes were treated with recombinant CaMKIV in the presence or absence of targeted-siRNA mediated down-regulation of CREB and mTOR. The ER stress markers, autophagy activation, mTOR/CREB signaling and insulin sensitivity were analyzed by western blotting or electron microscopy.

**Results:**

Treatment with CaMKIV significantly reversed tunicamycin-induced expression of p-PERK, cleaved-ATF6, Atg7 and LC3II. It also reduced p62 expression. In addition, levels of p-Akt and p-IRS-1 were increased. Moreover, CaMKIV inhibited activated ER stress and insulin resistance in Atg7 siRNA transfected adipocytes. However, the protective effects of CaMKIV on ER stress, insulin signaling, and autophagy function were nullified by suppression of mTOR or CREB in tunicamycin-treated adipocytes.

**Conclusion:**

This study proves recombinant CaMKIV inhibits tunicamycin-induced ER stress and insulin resistance by regulating autophagy. The protective effect of CaMKIV in adipocytes is affected at least partly through mTOR/CREB signaling. Our finding may offer novel opportunities for treating obesity and type 2 diabetes.

## Background

Insulin resistance is a complex pathological state of inappropriate cellular response to insulin hormone in insulin dependent cells, and it is a common risk factor in metabolic disorder associated diseases [1]. Adipose is now recognized as not only an energy-storage tissue, but also an endocrine tissue that can secrete a variety of bioactive substances including adipokines and proinflammatory cytokines [2]. Adipocytes and adipose tissue dysfunctions are believed to promote insulin resistance and lead to obesity [3, 4]. Although considerable progress has been made in understanding the molecular mechanisms underlying these individual disorders, satisfactory treatment modalities remain limited.

Abnormal autophagy has been implicated in a variety of diseases, such as obesity, type 2 diabetes, cancer and cardiovascular disease [5, 6]. The expression of *Atg7* in adipose tissue has a protective effect on insulin sensitivity in high-fat diet induced obesity, indicating autophagy activation contributes to the regulation of fat mass [7]. Autophagy was activated in adipose tissues of obese individuals and inhibition of autophagy enhanced pro-inflammatory gene expression both in adipocytes and adipose tissue explants, indicating autophagy might inhibit inflammatory gene expression in adipose tissue during obesity [8, 9]. Recently, autophagic dysfunction has been suggested a potential link to obesity and ER stress. There are three central ER stress signaling molecules in mammalian cells, namely IRE1α, PERK, and ATF6 [10]. Several strategies have been proposed to target ER stress as a therapeutic approach for pharmacological intervention in obesity and type 2 diabetes. Thus, downregulation of autophagy could be beneficial for adipocytes under ER stress and insulin resistance.

CaMKIV is a multifunctional serine/threonine protein kinase encoded by *CaMKIV* gene, and it plays a critical role in process of transcriptional regulation of lymphocytes, neurons and male germ cells [11–13]. Recently, CaMKIV has been identified as a regulator in glucose metabolism and insulin genes expression [14, 15], as well as in process of autophagy. For instance, CaMKIV not only increased autophagy to limit hepatic damage, but also involved in lipopolysaccharide induced inflammation and acute kidney injury [16, 17]. In the previous study, we further demonstrated CaMKIV limits metabolic damage through induction of hepatic autophagy by CREB in high-fat diet-induced obese mice [18]. As a significant regulator of autophagy, mTOR could be upregulated by CaMKIV [17]. In adipose tissues, autophagy was significantly increased in diabetes compared with non-diabetes, and mTOR expression was decreased in adipose of diabetes cases, indicating autophagy was negatively regulated by mTOR expression in adipose tissues of patients with diabetes [19].

In the previous study, to upregulate CaMKIV expression, constitutive active form of CaMKIV was usually used in vitro by transfection. However, in recent years, recombinant CaMKIV peptide has been used in several in vivo and in vitro studies [18, 20]. Exogenous CaMKIV peptides were suspected to transport intracellularly through binding to specific receptors. However, the membrane receptors of CaMKIV still remain unknown. Signaling through the transmembrane receptor Notch is widely used throughout animal development, and it is a major regulator of cell proliferation and differentiation [21]. It is interesting to note CaMKIV enhanced osteoclast differentiation through up-regulating Notch signaling [22]. In addition, it can potentiate Notch-dependent transcription by triggering nuclear export of SMRT (silencing mediator for retinoid and thyroid hormone receptor) [23]. These results gave us a clue that Notch might to be a potential receptor of CaMKIV.

Our previous study has demonstrated that CaMKIV plays an important role in regulating liver insulin sensitivity and plasma inflammation factors in high-fat diet-induced obese mice [18]. On the other hand, in white adipose tissues, CaMKK2 regulates adiposity and pre-adipocyte differentiation. In brown adipose tissues, CaMKK2 plays a critical role in adaptive thermogenesis [24]. Furthermore, CaMKIV is a direct downstream substrate of CaMKK2. Hence, we hypothesized CaMKIV might play an important role in regulating the metabolism of adipose tissue.

As a basic leucine zipper type transcription factor, CREB is ubiquitously expressed in organs. Its phosphorylation at Ser 133 is initiated by the recruitment of CaMKII and CaMKIV, interestingly, CaMKII can also phosphorylate CREB at Ser 142 and induce negative regulation [25, 26]. It has been suggested CREB regulates expression of IRE1a and PERK, which suggested CREB regulates the key components of UPR [27]. Rapamycin-induced autophagy against oxidative stress, synaptic/neurotransmission dysfunction, and cognitive deficits in the hippocampus of the rat brain through PI3K/Akt1-mTOR-CREB signaling pathway(s), which indicates mTOR/CREB signaling plays a critical role in autophagy function [28]. Therefore, we propose that CaMKIV could regulate mTOR/CREB signaling to inhibit ER stress and improve insulin sensitivity through reduction of autophagy in adipocytes.

This study was undertaken to test our hypothesis that CaMKIV through decreased autophagy can suppress ER stress and improve insulin resistance by mTOR/CREB signaling. We first tested the insulin sensitivity, ER stress and autophagy function in Tun (﻿tunicamycin)-treated mature 3 T3-L1 cells with or without recombinant CaMKIV. To further identify the mechanism of CaMKIV on insulin resistance, we next analyzed the markers of ER stress, autophagy and insulin sensitivity after blockage mTOR/CREB signaling in Tun-treated adipocytes. Our results provided a reciprocal functional interaction among CaMKIV, ER stress, autophagy and insulin signaling in Tun-treated adipocytes, indicating that CaMKIV regulated autophagy may function as an adaptive role in response to ER stress-induced insulin resistance.

## Methods

### Antibodies and reagents

The following antibodies were used: Atg7 (Cell Signaling, #2631), p62 (Cell Signaling, #5114), LC3 (Cell Signaling, #4108), CREB (Cell Signaling, #9197), p-CREB (Cell Signaling, #9198), mTOR (Cell Signaling, #2972), p-mTOR (Cell Signaling, #2971), IRS-1 (Cell Signaling, #2382), p-IRS-1 (Cell Signaling, #2381), Akt (Cell Signaling, #4685), p-Akt (Cell Signaling, #4060), p-PERK (Cell Signaling, #3179), PERK (Cell Signaling, #5683), cleaved-ATF-6 (Santa Cruz Biotechnology, #sc-166,659), GAPDH (Santa Cruz Biotechnology, #sc-47,724) and peroxidase goat anti-rabbit IgG (Santa Cruz Biotechnology, #sc-2768). Insulin were purchased from Sigma (Sigma-Aldrich, St. Louis, MO, USA). Mouse recombinant CaMKIV were obtained from Sino Biological (Sino Biological Inc. Wayne, PA, USA). Western Lightning Plus-ECL Enhanced Chemiluminescence Substrate Kit (PerkinElmer Inc., Richmond, CA, USA) was used to detect protein expression. Individual protein bands were quantified by ImageJ software. All other chemicals were obtained from standard resource and were of the highest grade available.

### Cell culture and treatment

3 T3-L1 were purchased from American Tissue Culture Collection (ATCC, Manassas, Virginia, USA, #ATCC® CL-173™) and maintained in DMEM (Gibco, Grand Island, New York, USA) with 10% fetal bovine serum (Gibco) at 37 °C in a humidified atmosphere with 5% CO_2_. The protocol of inducing maturation of 3T3L1 cells was performed as described [29] and which was mini-modified in our present study. In brief, for adipocytes differentiation, 100% confluent 3 T3-L1 were induced with MDI induction media (0.5 mM 1-methyl-3-isobutylmethylxanthine, 200 nM dexamethasone, 160 nM insulin, and DMEM with 10% FBS) (day 0). Two days later media was changed to 10% FBS/ DMEM with 160 nM insulin. Cells were then fed with this maintenance medium every 2 days. Full differentiation is usually achieved on the 12th day. The mature 3 T3-L1 adipocytes were used in our ongoing experiments. To induce ER stress, mature 3 T3-L1 cells were treated with different concentration (0-5 μg/ml) of Tun for 4 h. For the effects of CaMKIV, cells were treated with 100 ng/ml CaMKIV for 24 h. For blocking mTOR or CREB signaling, cells were transfected with 100 nM mTOR siRNA or 100 nM CREB siRNA for 24 h, respectively. For insulin signaling, cells were stimulated with 10 nM insulin for 10 min. Before each experiment, the medium was replaced by fresh medium.

### Electron microscopy analysis

The protocol we followed was described previously [30]. In brief, we fixed adipocytes in 4% paraformaldehyde/2% glutaraldehyde/0.1 M sodium cacodylate pH 7.3, post-fixed in 1% osmium tetraoxide and embedded in epoxy resin (Epon). Then the ultrathin sections (80 nm) were stained by aqueous uranyl acetate, and lead citrate and examined with JEOL 2000FX transmission electron microscope (JEOL)., The numbers of autophagolysosomal-like vacuoles were counted in each field and normalized by the surface area for quantification of autophagolysosome-like vacuoles.

### Small interfering RNAs (siRNAs) and transfection

Small interfering RNA (siRNA) for target genes (Atg7: sc-41,448; CREB: sc-35,111; mTOR: sc-35,410, Santa Cruz Biotechnology, Inc., Dallas, TX, USA) or scrambled siRNA (CREB, sc-37,007, Santa Cruz Biotechnology, Inc., Dallas, TX, USA) were using Lipofectamine® RNAiMAX Transfection Reagent (Invitrogen, Carlsbad, CA, USA) according to the manufacturer’s protocol. The transfected cells were cultured in medium containing 10% FBS for 24 h after transfection. The knockdown efficiency was assessed by western blot.

### Western blotting and immunoprecipitation

To prepanre cell lysates, RIPA Lysis and Extraction Buffer (Invitrogen; ThermoFisher Scientific, Inc., MA, USA) which contained 10% protease inhibitor (Thermo Scientific, USA) were used to extract cell lysates by incubated on ice for 30 min, and then centrifuged at 14000 x g for 15 min at 4 °C. The protein concentrations were determined by BCA kit (Thermo Scientific, USA). For western blotting, we used the protocol which was performed previously [31]. In brief, we first mixed supernatants with 4x SDS-PAGE sample loading buffer, and they were denatured at 95 °C for 10 min. Second, the proteins were separated by SDS-PAGE gel, transferred to a polyvinylidene difluoride membranes, incubated with specific primary antibodies at 4 °C overnights, and detected with horseradish peroxidase (HRP)-conjugated secondary antibodies by using a VersaDoc Image System (BioRad, Hercules, CA, USA). For immunoprecipitation, the lysate was treated using the Dynabeads™ Protein G Immunoprecipitation Kit (Invitrogen; ThermoFisher Scientific, Inc., MA, USA) according to the protocol. The final precipitated proteins were analyzed via western blotting with the corresponding antibodies.

### Statistics

Data were analyzed by the Prism software, version 8.0 (GraphPad Software Inc., San. Diego, CA, US). Characteristics of subjects between 2 groups was performed using Mann Whitney test. Multiple comparisons of quantitative variables among groups were made using Kruskal Wallis test [32]. Data were presented as mean ± SD. N represents the number of animals used. A *P* value of<0.05 or *P* value of<0.01 was considered as significantly or highly significantly difference.

## Results

### Tun-mediated ER stress increases autophagy in mature 3 T3-L1 adipocytes

**Fig. 1:**
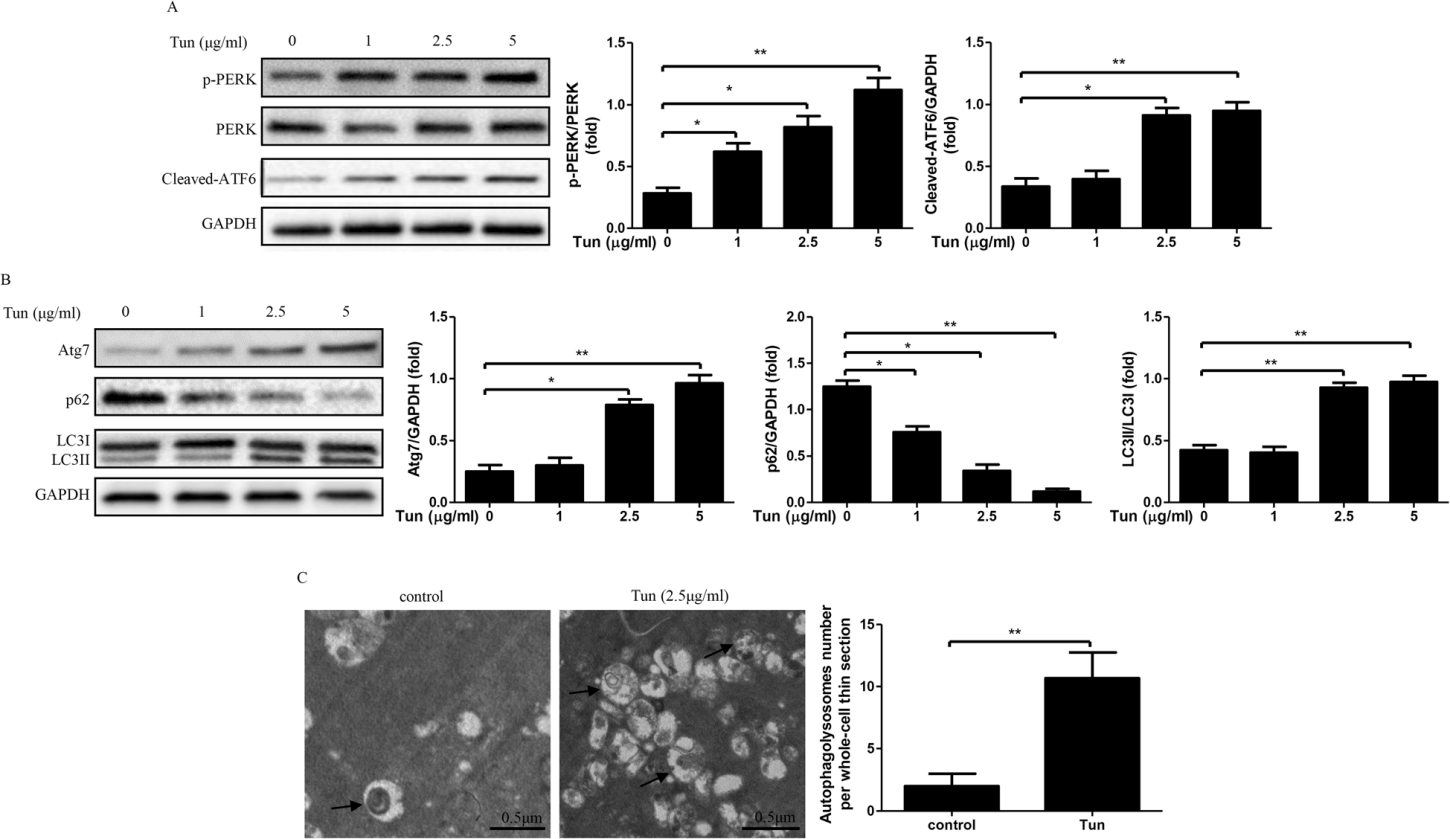
Induction of autophagic dysfunction due to ER stress induced by tunicamycin in adipocytes treated with 0, 1, 2.5 or 5 μg/ml Tun for 4 h. All indicators were measured at protein level. The relative quantity of protein was analyzed using Quantity One software. **a** ER stress markers including PERK phosphorylation (p-PERK) and cleaved-ATF6 in adipocytes. **b** Autophagy-related proteins Atg7, p62 and LC3 in adipocytes treated with 2.5 μg/ml Tun for 4 h. **c** Representation electron micrographs of adipocytes treated with 2.5 μg/ml Tun for 4 h. Quantification of autophagolysosome-like vacuoles per field in the EM images, Scale bars, 0.5 μm. Quantitative data are presented as means ± SD from at least 3 independent experiments. * *P* < 0.05 or ** *P* < 0.01

To determine the effects of pharmacological ER stress on autophagic function, the mature 3 T3-L1 adipocytes were treated with various dose of Tun (0-5 μg/ml) for 4 h, then the ER stress and autophagy markers were examined. The data suggested phosphorylation of PERK expressions and cleaved-ATF6 expressions were increased significantly after Tun exposure with concentration of ≧2.5 μg/ml compared with the control group (Fig. 1a). Moreover, we observed autophagy markers such as Atg7 and LC3II expressions were markedly increased, as well as decreased p62 expressions after 4 h of 2.5 and 5 μg/ml Tun treatment compared with control group (Fig. 1b). Hence, we selected 2.5 μg/ml Tun to induce ER stress autophagic dysfunction in the ongoing experiments. In addition, the results of EM examination in mature 3 T3-L1 adipocytes demonstrated a significant induction of autophagosome/ autolysosome formation in adipocytes treated with 2.5 μg/ml Tun for 4 h compared with control (Fig. 1c). These results suggest that Tun-induced ER stress mediates autophagic dysfunction.

### CaMKIV reverses Tun-induced ER stress and autophagic dysfunction and improves impaired insulin sensitivity in adipocytes

**Fig. 2:**
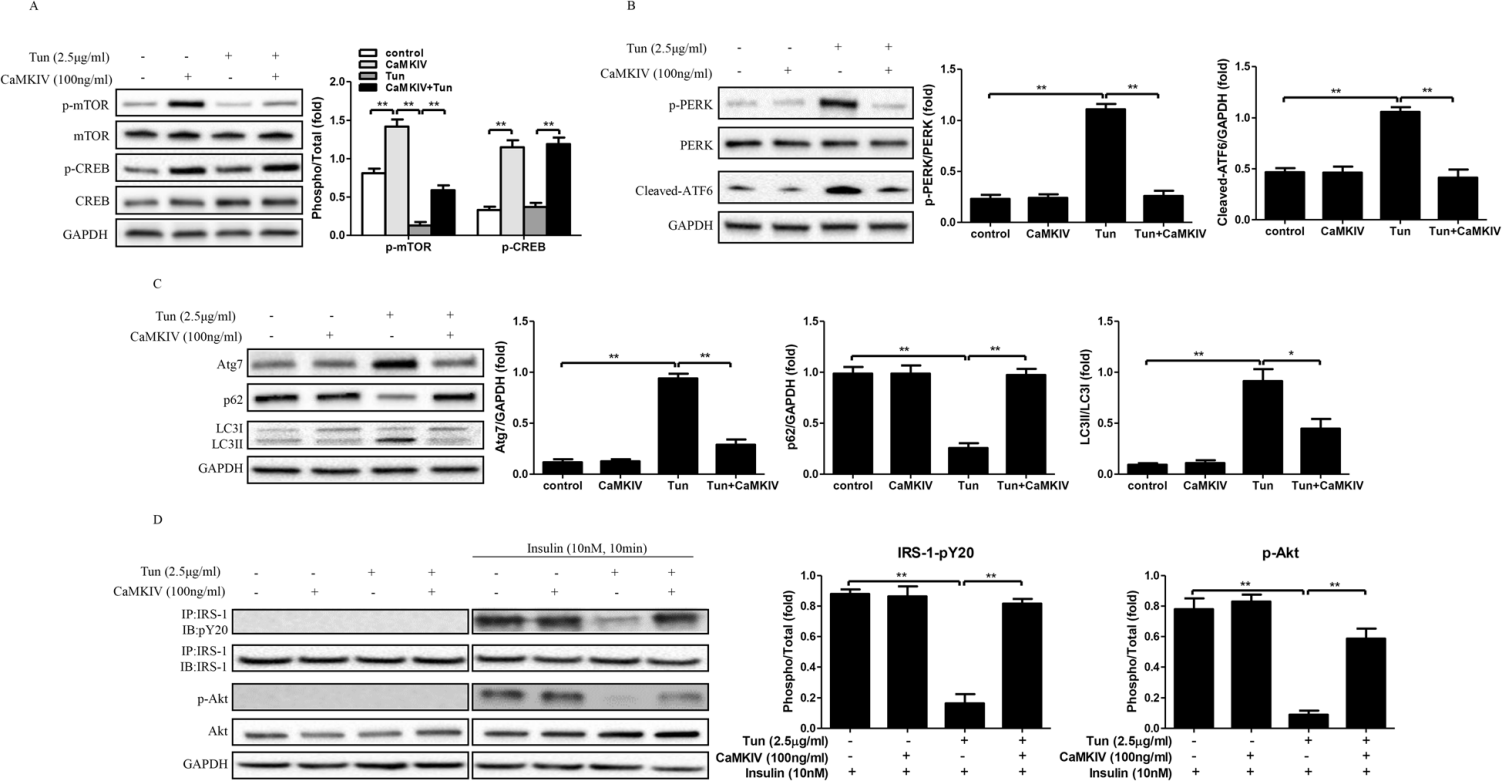
Recombinant CaMKIV incubation reduces ER stress-induced insulin resistance and autophagy dysfunction in mature adipocytes. Adipocytes were pretreated with Tun (2.5 μg/ml) for 4 h, followed by CaMKIV (100 ng/ml) for 24 h. For insulin signaling, cells incubated in the absence or presence of 10 nM insulin for 10 min. All indicators were measured at protein level. The relative quantity of protein was analyzed using Quantity One software. **a** p-mTOR, p-CREB expression and their total protein levels. **b** ER stress markers including PERK phosphorylation (p-PERK) and cleaved-ATF6 in adipocytes. **c** Autophagy-related proteins Atg7, p62 and LC3 in adipocytes. **d** IRS-1 tyrosine phosphorylation (pY), Akt serine 473 phosphorylation (p-Akt), and their total protein levels were examined in mice adipocytes either with IP followed by IB or by direct immunoblotting. Quantitative data are presented as means ± SD from at least 3 independent experiments. IB, immunoblotting; IP, immunoprecipitation. * *P* < 0.05 or ** *P* < 0.01

To understand the effects of CaMKIV on autophagy and insulin signaling, the ER stress, autophagy and insulin signaling markers were evaluated. Mature 3 T3-L1 adipocytes were pretreated with 2.5 μg/ml Tun for 4 h to induce ER stress and autophagic dysfunction. As the results shown, p-mTOR expression was inhibited by Tun treatment. However, CaMKIV not only induced phosphorylated CREB after CaMKIV incubation with or without Tun pretreated, but also increased the expression of p-mTOR (Fig. 2a). These data suggested CaMKIV regulates phosphorylation of mTOR and phosphorylated CREB expressions in Tun-treated adipocytes. In addition, Tun treatment not only elevated the expression of phosphorylation of PERK and cleaved-ATF6, but also induced autophagy, as evidenced by increased Atg7 and LC3-II expression and decreased of p62 expression in adipocytes (Fig. 2b and c). Meanwhile, insulin signaling was impaired in Tun-treated cells, which was identified by the reduction of Akt phosphorylation and IRS-1 tyrosine phosphorylation (Fig. 2d). Remarkably, Tun and CaMKIV co-treated adipocytes displayed a reduced PERK phosphorylation and cleaved-ATF6 expression. Meanwhile, the increased Atg7 and LC3-II expressions were reversed by CaMKIV in Tun-pretreated adipocytes, as well as the decreased p62 expressions (Fig. 2b and c). Additionally, the recovery of insulin sensitivity in CaMKIV-treated adipocytes with Tun treatment was also evident, as demonstrated by increased Akt phosphorylation and IRS-1 tyrosine phosphorylation (Fig. 2d). These findings indicate CaMKIV reverses Tun-induced autophagic dysfunction and ER stress, restores insulin signaling and regulates mTOR and CREB expression in vitro.

### CaMKIV inhibits ER stress and reverses insulin resistance in Atg7 siRNA transfected adipocytes

**Fig. 3:**
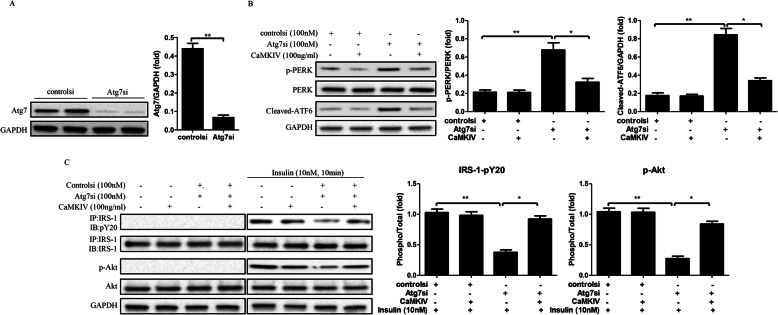
CaMKIV rescues autophagy dysfunction induced ER stress and insulin resistance in mature adipocytes. Adipocytes were pretreated with 100 nM Atg7 siRNA (Atg7si) or control siRNA (controlsi) for 24 h, followed by 2 mM CaMKIV for 24 h. For insulin signaling, cells incubated in the absence or presence of 10 nM insulin for 10 min. All indicators were measured at protein level. The relative quantity of protein was analyzed using Quantity One software. **a** Total protein was isolated from siRNA adipocytes and immunoblotted with antibodies for Atg7. **b** Effect of CaMKIV on ER stress in Atg7^−/−^ adipocytes. **c** IRS-1 tyrosine phosphorylation (pY) and Akt serine 473 phosphorylation (p-Akt) in Atg7si transfected adipocytes with or without CaMKIV. Quantitative data are presented as means ± SD from at least 3 independent experiments. IB, immunoblotting; IP, immunoprecipitation. * *P* < 0.05 or ** *P* < 0.01

Autophagy activation plays an important role in regulating ER stress and insulin resistance [33]. To further clarify the effects of CaMKIV on ER stress and insulin resistance, we used to build an autophagic dysfunction adipocytes model. Adipocytes were transfected with 100 nM Atg7si for 24 h, which was validated by a reduced Atg7 protein expression (Fig. 3a). Notably, downregulation of Atg7 significantly increased phosphorylation of PERK and cleaved-ATF6 expression, as well as decreased the phosphorylation of IRS-1 and Akt (Fig. 3b and c), indicating defective autophagy can induce ER stress and insulin resistance. However, CaMKIV treatment inhibited ER stress and improved impaired insulin sensitivity in Atg7si-transfected adipocytes, as demonstrated by reduced PERK phosphorylation and cleaved-ATF6 expression, and by increased phosphorylation of IRS-1 and Akt (Fig. 3b and c). These results not only demonstrate the importance of functional autophagy in maintaining cellular homeostasis, but further indicate CaMKIV reduced ER stress and improved insulin signaling in adipocytes of autophagic dysfunction.

### CaMKIV restores insulin sensitivity and autophagic dysfunction through mTOR/CREB signaling in Tun-treated adipocytes

**Fig. 4:**
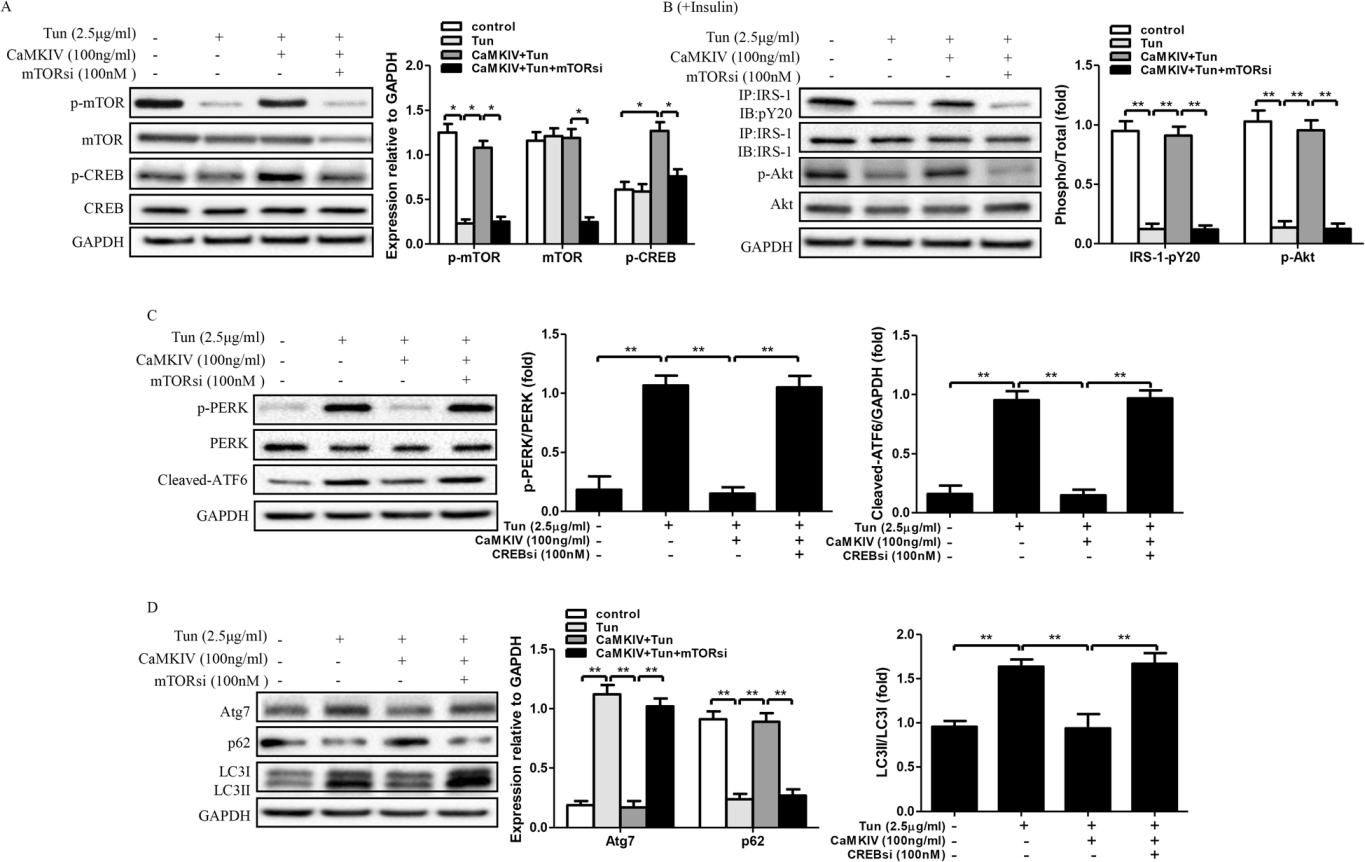
Effect of CaMKIV on ER stress, insulin signaling, and autophagy in mTOR siRNA transfected adipocytes. Cells were cultured with Tun (2.5 μg/ml) for inducing insulin resistance. Cells were cultured in the presence or absence of CaMKIV with or without 100 nM mTOR siRNA (mTORsi) for 24 h. For insulin signaling, cells were stimulated with 10 nM insulin for 10 min. All indicators were measured at protein levels. The relative quantity of proteins was analyzed using Quantity One Software. **a** p-mTOR, p-CREB expression and their total protein levels. **b** IRS-1 tyrosine phosphorylation (pY), Akt serine 473 phosphorylation (p-Akt), and their total protein levels were examined in mice adipocytes either with IP followed by IB or by direct immunoblotting. **c** ER stress markers including PERK phosphorylation (p-PERK) and cleaved-ATF6 in adipocytes. **d** Autophagy-related proteins Atg7, p62 and LC3 in adipocytes. Quantitative data are presented as means ± SD from at least 3 independent experiments. * *P* < 0.05 or ** *P* < 0.01

**Fig. 5:**
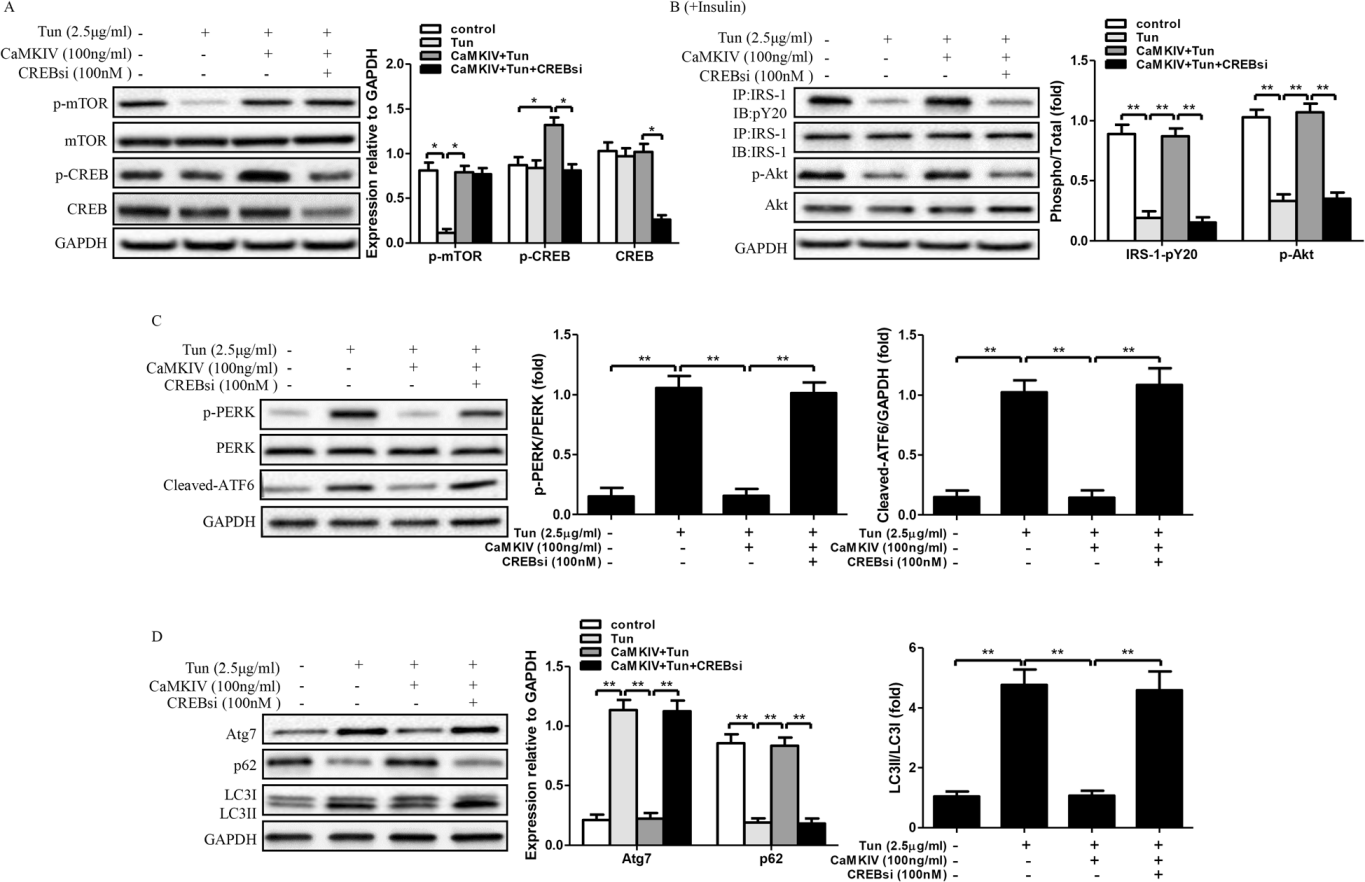
Effect of CaMKIV on ER stress, insulin signaling, and autophagy in CREB siRNA transfected adipocytes. Cells were cultured with Tun (2.5 μg/ml) for inducing insulin resistance. Cells were cultured in the presence or absence of CaMKIV with or without 100 nM CREB siRNA (CREBsi) for 24 h. For insulin signaling, cells were stimulated with 10 nM insulin for 10 min. All indicators were measured at protein levels. The relative quantity of proteins was analyzed using Quantity One Software. **a** p-mTOR, p-CREB expression and their total protein levels. **b** IRS-1 tyrosine phosphorylation (pY), Akt serine 473 phosphorylation (p-Akt), and their total protein levels were examined in mice adipocytes either with IP followed by IB or by direct immunoblotting. **c** ER stress markers including PERK phosphorylation (p-PERK) and cleaved-ATF6 in adipocytes. **d** Autophagy-related proteins Atg7, p62 and LC3 in adipocytes. Quantitative data are presented as means ± SD from at least 3 independent experiments. * *P* < 0.05 or ** *P* < 0.01

Our results demonstrate CaMKIV inhibits ER stress and improves insulin sensitivity through recovery of autophagy. But the underlying mechanism still remain unclear. It has been identified CREB, an important transcriptional factor, involved in CaMKIV-mediated autophagy in hepatic ischemia-reperfusion injury [16]. Further, CaMKIV regulates autophagy through mTOR signaling in lipopolysaccharide-induced inflammation and acute kidney injury [17]. Therefore, we suspected that the protective effects of CaMKIV on autophagy, ER stress and insulin sensitivity through mTOR/CREB signaling. To clarify our hypothesis, we then cultured adipocytes in presence of Tun and/or CaMKIV, with or without the target-siRNA of each signaling pathway such as mTOR siRNA (mTORsi) and CREB siRNA (CREBsi). As the results shown in Fig. 4a, CaMKIV treatment not only increased the expression of phosphorylated CREB, but also significantly improved the expression of p-mTOR which reduced by Tun. Meantime, mTORsi transfection not only reduced the expressions of p-mTOR and total mTOR, but also decreased the expression of p-CREB in Tun and CaMKIV incubated cells. Moreover, Tun treatment markedly decreased insulin sensitivity and induced ER stress and autophagy in adipocytes (Fig. 4b-d). However, CaMKIV can suppress ER stress, inhibit autophagy, and improve insulin sensitivity in Tun-pretreated cells. But blockade of mTOR signaling can nullified the protective effects of CaMKIV (Fig. 4b-d). We next detected the role of CREB in the protective process of CaMKIV. The results suggested CREBsi transfection inhibited phosphorylated CREB and total CREB expressions, but it did not affect the expressions of phosphorylated mTOR and Total mTOR (Fig. 5a). Furthermore, downregulation of CREB can block the protective effects of CaMKIV on ER stress, autophagy activation, and insulin signaling in Tun and CaMKIV co-treated adipocytes, suggesting CaMKIV inhibits ER stress, suppresses autophagy, and improves impaired insulin signaling through phosphorylation of CREB. Of note, these results indicate the protective role of CaMKIV on ER stress, autophagy and insulin signaling through mTOR/CREB signaling in adipocytes (Fig. 5b-d).

## Discussion

In this study, we first proved recombinant CaMKIV protein inhibites autophagy and ER stress and improves insulin sensitivity in tunicamycin-treated mature 3 T3-L1 adipocytes. Next, we further identified these protective effects of CaMKIV were nullified by downregulating mTOR or CREB expression, indicating CaMKIV regulates ER stress, abnormal autophagy and insulin sensitivity in Tun-treated 3 T3-L1 cells through mTOR/CREB signaling. In addition, CaMKIV inhibits ER stress and improves insulin sensitivity in Atg7 siRNA transfected cells. This result further demonstrated the protective effect of CaMKIV on autophagy, ER stress and insulin signaling.

Recently, autophagy dysfunction and ER stress are recognized as the important factors of insulin resistance [33]. In the process of ER stress, ER stress sensors IRE1, PERK and ATF6 are activated, leading to a series of downstream events, including reducing translation and increasing transcription ER chaperones to make sure that normal cell function and viability are maintained [34]. Autophagy is an evolutionarily conserved lysosomal mechanism that enable cells to conserve and maintain cellular biomass quality and quantity by targeting damaged or unused proteins and even organelles of degradation [6]. Previous studies have reported ER stress can be triggered by obesity or metabolic factors, such as lipids, glucose and cytokines [35–37], and it was a common factor in high-fat feeding, genetic obesity and elderly [37, 38]. These results demonstrated autophagic dysfunction and ER stress were the main pathway that response to the pathological factor, including lipotoxicity, inflammation and insulin resistance. In the present study, Tun treatment not only induces ER stress, but also induces autophagic dysfunction and insulin resistance in adipocytes. Our data further provided the evidence that autophagy remarkably associated with ER stress and insulin resistance.

CaMKIV has been identified as a regulator in glucose metabolism and insulin signaling. For instance, its overexpression in skeletal muscle led to systemic improvements in insulin sensitivity and its activation involved in hepatic and adipose insulin action via increases in myokines released from the skeletal muscle [39]. Moreover, 12-week of CaMKIV injection in obese mice could improve high-fat diet-induced hepatic insulin resistance, further indicating CaMKIV plays an important role in whole-body glucose metabolism and hepatic insulin signaling [18]. It is well-known that Ca^2+^ signaling is a major regulator of CaMKIV in cellular, and disruption of Ca^2+^ homeostasis in the ER is well documented to trigger ER stress. According for these finding, we suspected CaMKIV might affect ER stress. Our results demonstrated CaMKIV inhibits ER stress markers, such as PERK and Cleaved-ATF6, indicating CaMKIV plays a critical role in regulating ER function.

Recent evidences implicate CaMKIV involved in autophagy regulation. However, its effect on autophagy activation is opposite in various tissues. John Evankovich and their colleagues [16] suggested CaMKIV limited organ damage in hepatic ischemia-reperfusion (I/R) injury through induction of autophagy. Compared with wild-type mice, the expressions of LC3II and Beclin 1 were significantly decreased in CaMKIV KO mice after I/R. The in vitro study also demonstrated upregulation of CaMKIV by transfecting with dominant-active mutant CaMKIV-dCT could increase LC3II expression. Another study showed Atg7 and LC3II expressions were significantly induced in pMФ which were transfected with constitutively active CaMKIV (CaMKIV-dCT), indicating CaMKIV increases autophagy activity. Interestingly, elevating CaMKIV activity through the transfection of active CaMKIV-dCT increased mTOR protein concentration, suggesting CaMKIV increases autophagy through inducing mTOR expression [17]. In our previous study, high-fat diet-induced defective liver autophagy was improved by long-term recombinant CaMKIV protein injection in obese mice [18]. These results indicated CaMKIV have a pro-autophagic effect in liver. Nevertheless, autophagy was significantly induced in adipose tissue of diabetes compared with non-diabetes, as well as decreased mTOR expressions in adipose tissue of diabetes cases. However, LC3 expression significantly increased after rapamycin (an inhibitor of mTOR) treatment with adipocytes, indicating autophagy was negatively regulated by mTOR expression in adipose tissues [19]. mTOR typically serves as a negative regulator of autophagy, and as a consequence, initiation of autophagy is largely dependent on release of mTOR inhibition [40]. Our data suggested CaMKIV incubation significantly increased the p-mTOR expression, suggesting CaMKIV inhibited autophagy associated with p-mTOR expression. Hence, CaMKIV not only can increase autophagy but also can decrease autophagy in different tissues might be due to regulate mTOR signaling pathway.

Recent studies strongly suggested several factors including metabolic stressors, obesity, free fatty acid, and inflammatory cytokines could promote autophagic disorder of adipocytes [41, 42]. Targeted deletion of the *Atg7* gene in adipose tissue can destroy autophagy pathway, which protects mice from high-fat diet-induced obesity and insulin resistance, suggesting that activation of the autophagy-mediated pathway may be one of the mechanisms of obesity-induced insulin resistance [7]. In our study, *Atg7* ablation-induced ER stress and impaired insulin sensitivity could be reversed by CaMKIV incubation, suggesting the protective role of CaMKIV in autophagy defective adipocytes.

CREB is a transcription factor that integrates growth factors, Ca^2+^, and cyclic AMP-induced signaling [43]. As a target of the cAMP/PKA pathway, CREB can be activated by Ca^2+^/calmodulin-dependent protein kinase and phosphorylated by kinases of the MAPK pathway [44]. Several groups subsequently showed that CaMKIV phosphorylated CREB at Ser133 in vitro and stimulated CREB transcriptional activity in vivo, which led to the suggestion that CaMKIV was the principal Ca^2+^-stimulated CREB kinase [26, 45]. The mTOR/CREB pathway is an intracellular signaling pathway, and it is important in several normal cellular function and in regulation of autophagy [46, 47]. Here, we supposed that the activation of mTOR/CREB signaling is required for CaMKIV-mediated ER stress, autophagy, and the restoration of insulin signaling. It is interesting to note activated CREB has been demonstrated in adipose cells under obese conditions, where it promotes insulin resistance by triggering expression of ATF3 and downregulating expression of GLUT4, indicating CREB plays and negative role in obesity induced insulin resistance [48]. However, in our study, ablation of CREB nullified the protective role of CaMKIV in regulation of autophagy, ER stress and insulin resistance. Our study enhances understanding of the mechanisms by which mTOR/CREB contributes to the regulation of CaMKIV-induced modulation in adipose.

This study demonstrated for the first time that the inhibition of ER stress and improvement of insulin signaling is due to the restored autophagy function which improved by recombinant CaMKIV protein rather than the direct effects of this enzyme. However, our study does have limitations which should be further investigated. First of all, it has not been shown the effects of transfection-mediate overexpression of CaMKIV on ER stress, autophagy and insulin signaling in mature adipocytes. Therefore, we cannot be certain that whether the in vitro treatment by CaMKIV protein and transfection-mediate overexpression of CaMKIV produce the same effects on ER stress, autophagy and insulin signaling in these adipocytes or not. On the other hand, although we proposed the protective effects of CaMKIV in adipocytes might be due to intracellular signaling, the receptors of CaMKIV have not been identified, as well as the binding sites. Hence, our further prospective studies are needed to demonstrate the effects of transfection-mediate overexpression of CaMKIV on ER stress, autophagy and insulin sensitivity in adipocytes, and to find the specific membrane receptors of CaMKIV and the binding sites. Although further studies are required, our results provided therapeutic implications of CaMKIV for modifying insulin signaling and autophagy function under the condition of ER stress in the adipocytes.

## Conclusion

Recombinant CaMKIV protein inhibits ER stress and improves impaired insulin sensitivity by restoration of autophagy in mature 3 T3-L1 adipocytes, and the protective effects of CaMKIV on autophagy, ER stress and insulin signaling through regulating mTOR/CREB signaling. Our study contributes to elucidating the potential role of CaMKIV in the pathogenesis of obesity and type 2 diabetes.

## Supplementary information

**Additional file 1.**

## Data Availability

The data generated or analyzed during this study are included in this article.

## Abbreviations

Akt: Protein kinase B
ATF6: Activation of transcription factor 6
Atg7: Autophagy-related 7
CaMKII: Calcium/calmodulin-dependent protein kinase II
CaMKIV: Calcium/calmodulin-dependent protein kinase IV
CREB: Cyclic AMP-responsive element binding protein
ER: Endoplasmic reticulum
GAPDH: Glyceraldehyde-3-phosphate dehydrogenase
IgG: Immunoglobulin
IRE1α: Inositol-requiring enzyme 1α
IRS: Insulin receptor substrate
LC3: Microtubule associated protein 1 light chain 3
mTOR: mammalian target of rapamycin
p: phosphorylated
PERK: PKR-like ER eIF2α kinase
PI3K: Phosphoinositide 3-kinase
siRNA: small interfering RNA
UPR: Unfolded protein response
