# Supplementary figures and images for: In vitro treatment of 3 T3-L1 adipocytes with recombinant Calcium/calmodulin-dependent Protein Kinase IV (CaMKIV) limits ER stress and improves insulin sensitivity through inhibition of autophagy via the mTOR/CREB signaling pathway
Source: BMC Endocr Disord. 2020 Jul 13;20:104. doi: 10.1186/s12902-020-00589-2 (PMC7359471; doi:10.1186/s12902-020-00589-2)

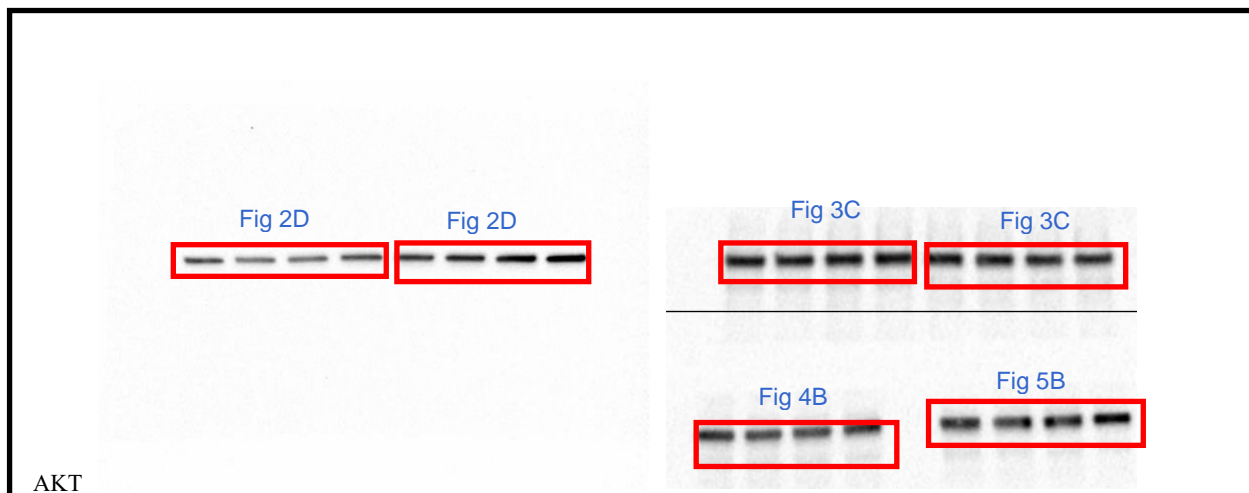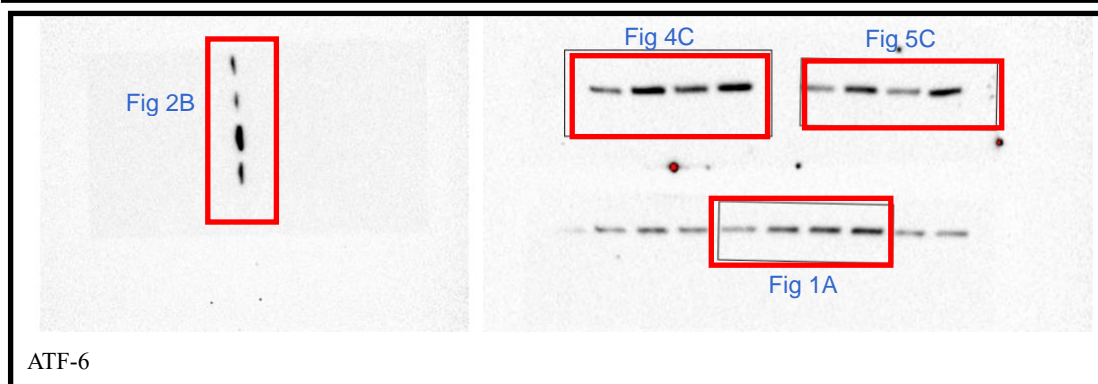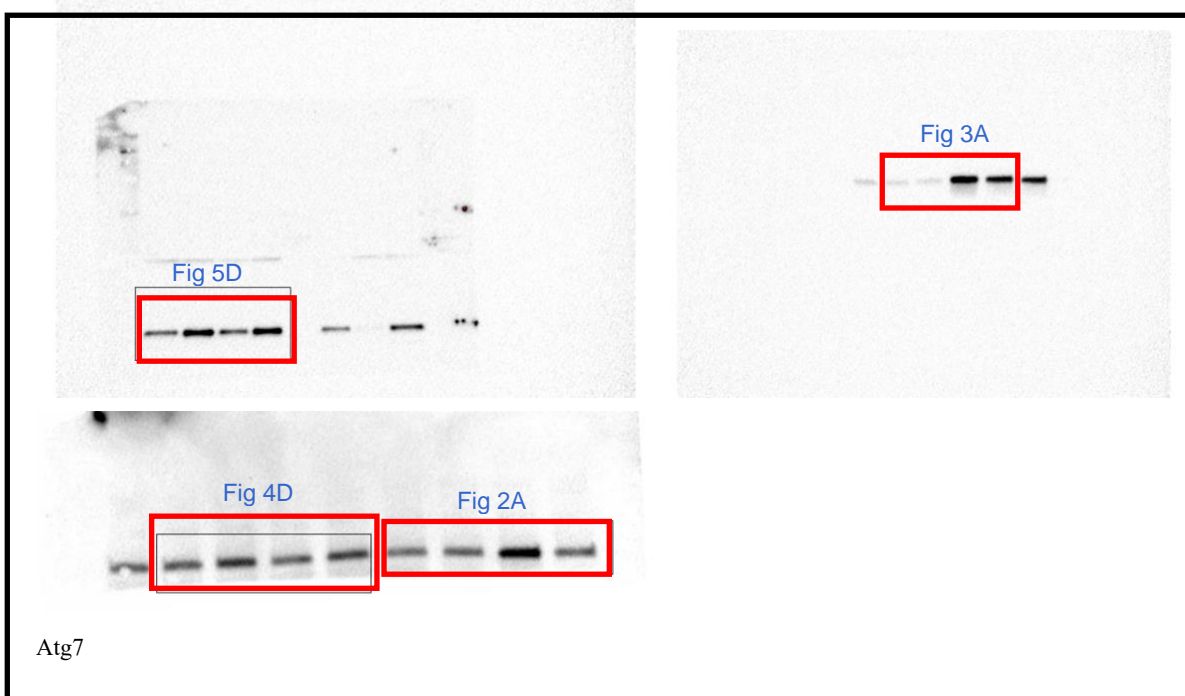

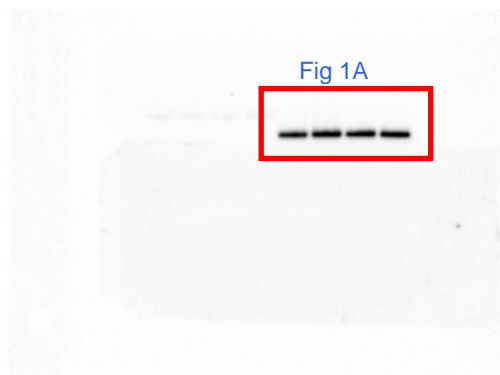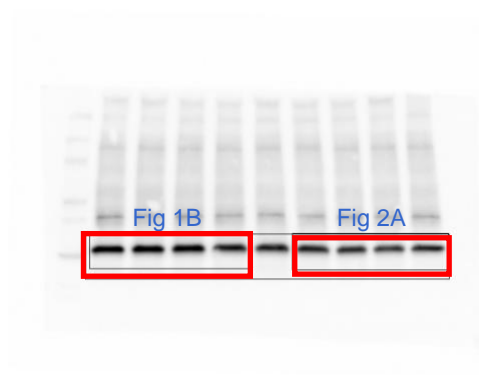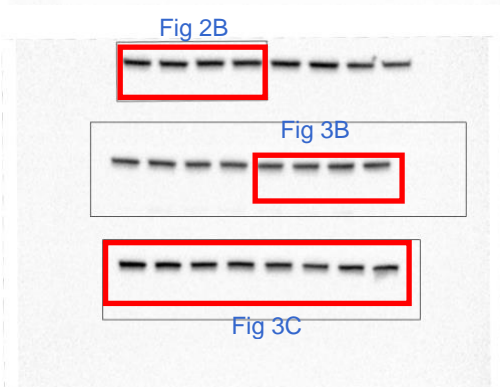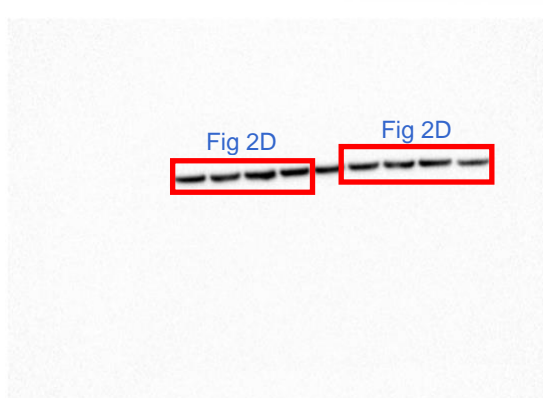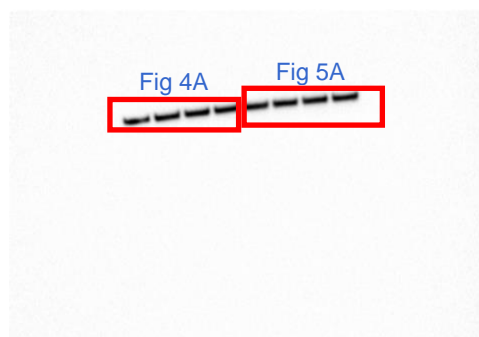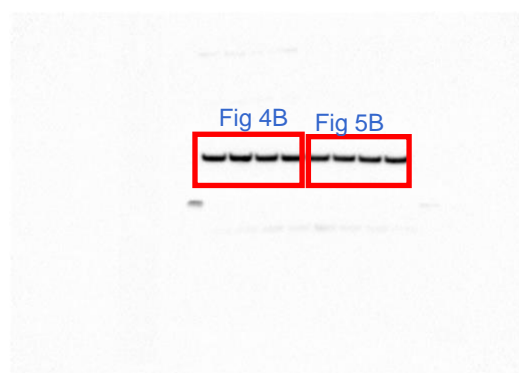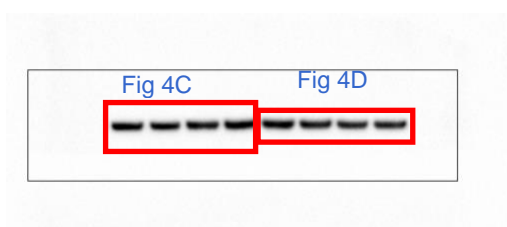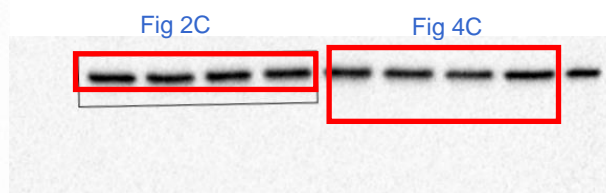

GAPDH

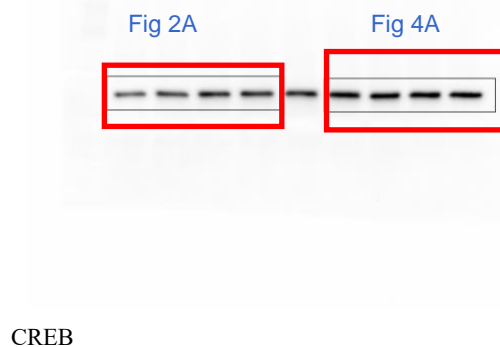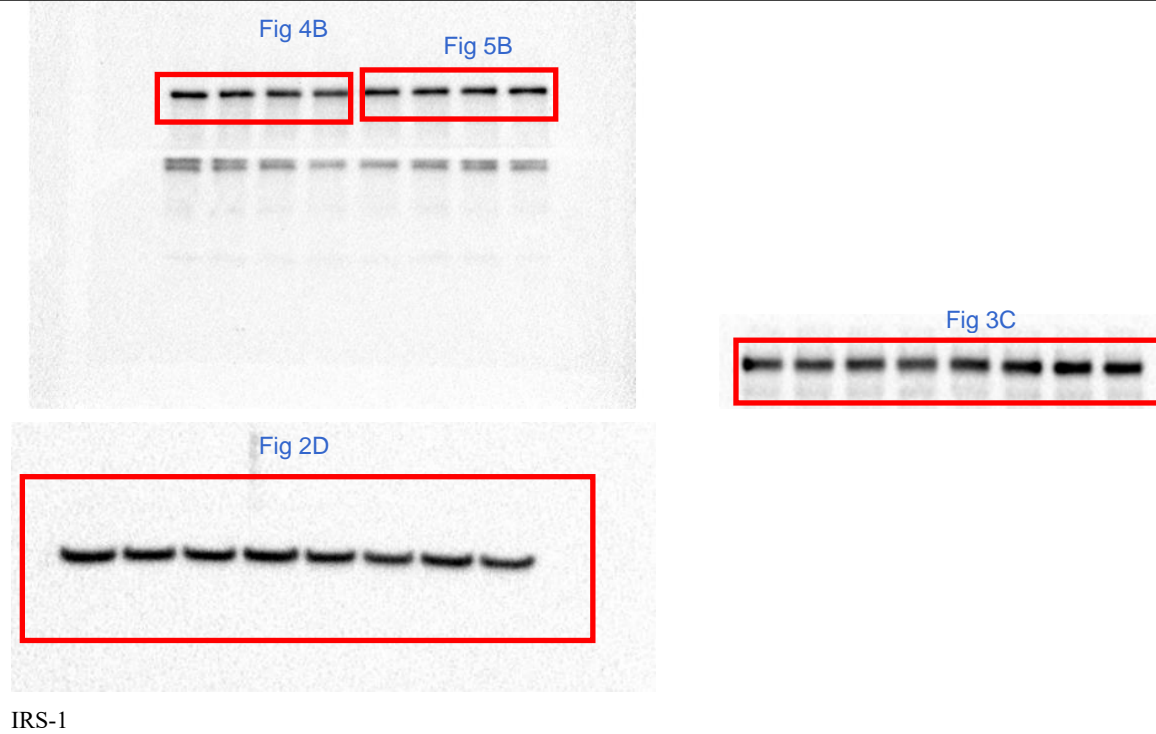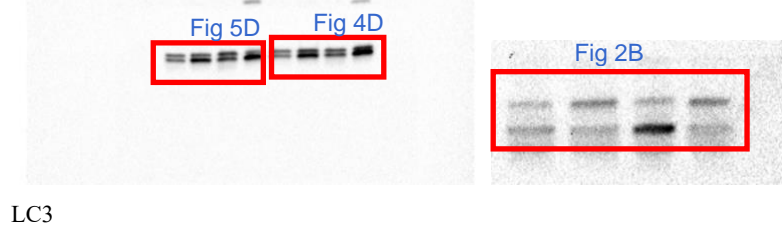

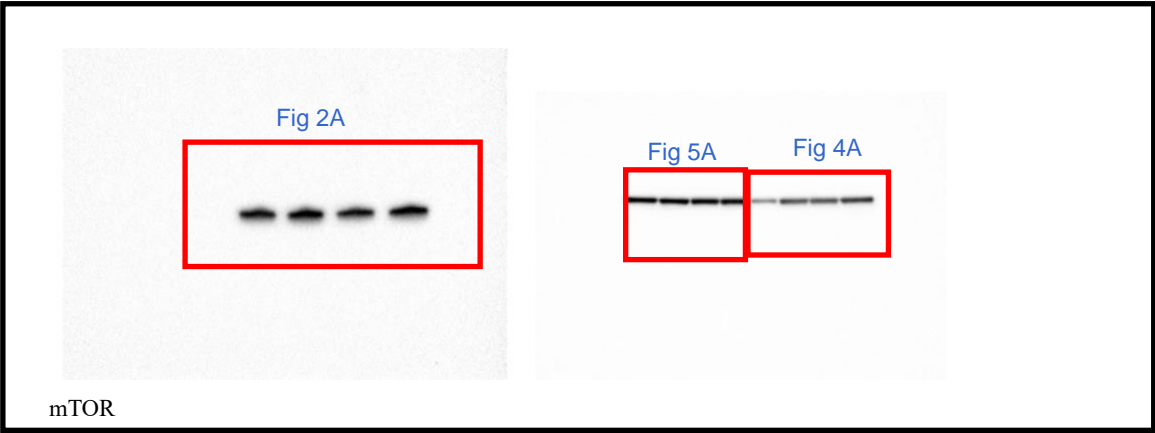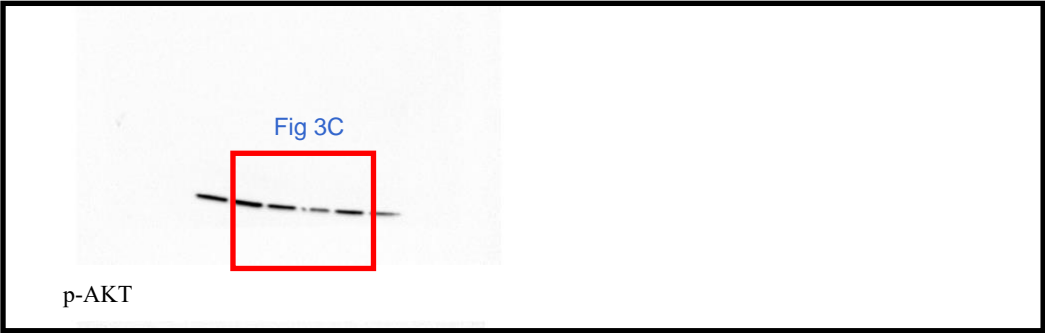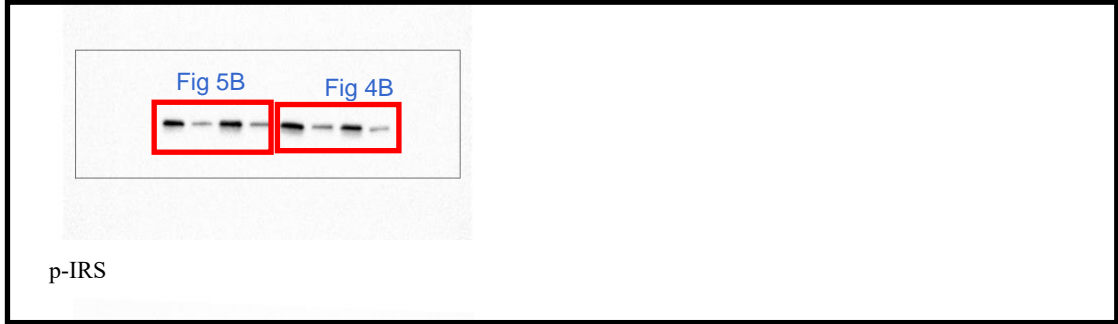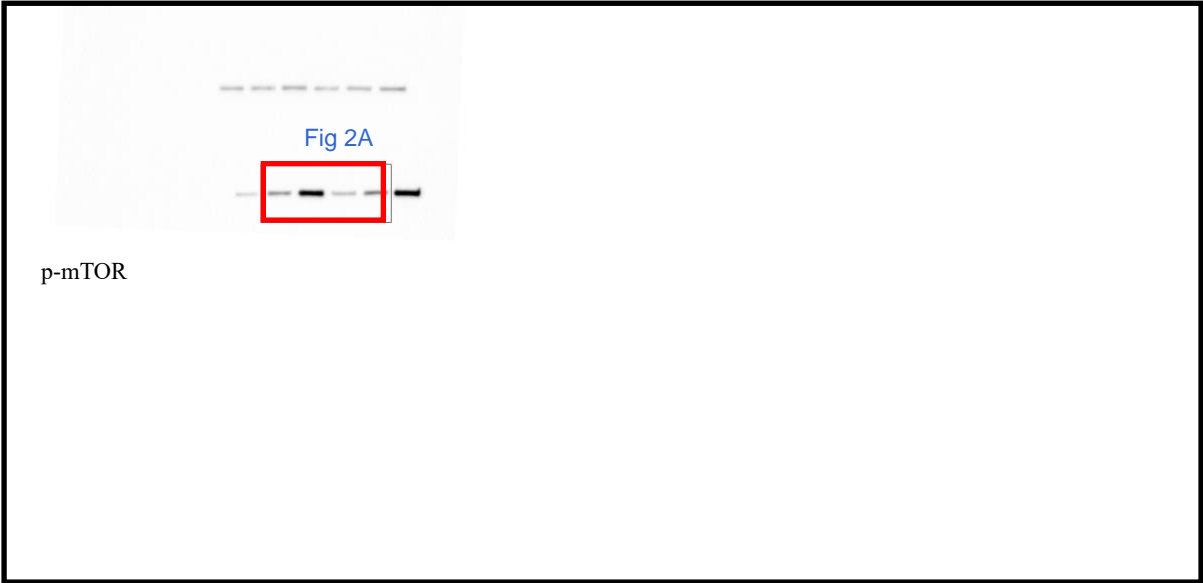

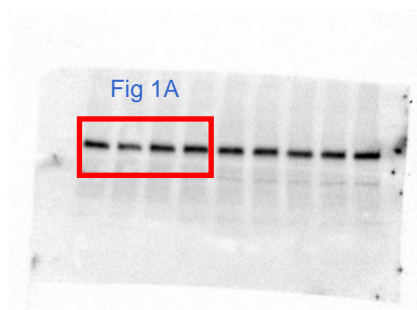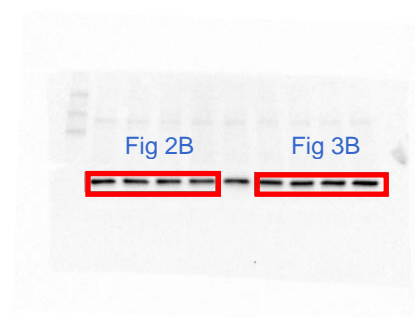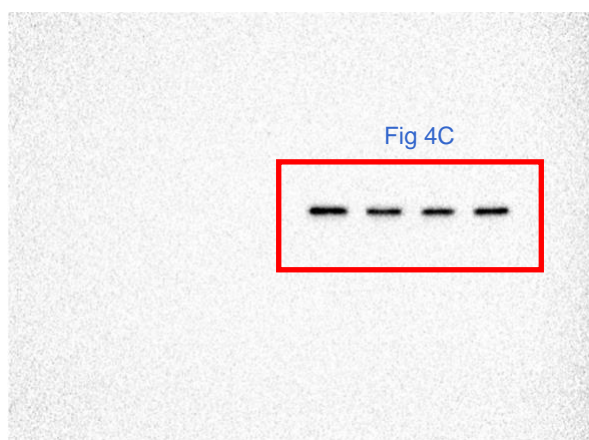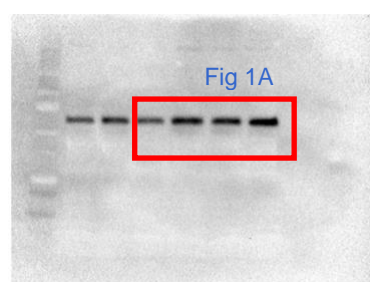

PERK

Supplement: Supplementary file 1 — Additional file 1. [file 12902_2020_589_MOESM1_ESM.pdf]
